# Evolutionary acquisition of promoter-associated non-coding RNA (pancRNA) repertoires diversifies species-dependent gene activation mechanisms in mammals

**DOI:** 10.1186/s12864-017-3662-1

**Published:** 2017-04-07

**Authors:** Masahiro Uesaka, Kiyokazu Agata, Takao Oishi, Kinichi Nakashima, Takuya Imamura

**Affiliations:** 1 Department of Stem Cell Biology and Medicine, Graduate School of Medical Sciences, Kyushu University, Fukuoka, 812-8582 Japan; 2 Department of Biophysics and Global COE Program, Graduate School of Science, Kyoto University, Kyoto, 606-8502 Japan; 3 Department of Cellular and Molecular Biology, Primate Research Institute, Kyoto University, Aichi, 484-8506 Japan; 4 Department of Biological Sciences, Graduate School of Science, The University of Tokyo, Tokyo, 113-8654 Japan; 5 Department of Life Science, Faculty of Science, Graduate Course in Life Science, Graduate School of Science, Gakushuin University, Tokyo, 171-8588 Japan

**Keywords:** Long non-coding RNA, Species diversity, Epigenetic regulation, Evolution

## Abstract

**Background:**

Recent transcriptome analyses have shown that long non-coding RNAs (ncRNAs) play extensive roles in transcriptional regulation. In particular, we have reported that promoter-associated ncRNAs (pancRNAs) activate the partner gene expression via local epigenetic changes.

**Results:**

Here, we identify thousands of genes under pancRNA-mediated transcriptional activation in five mammalian species in common. In the mouse, 1) pancRNA-partnered genes confined their expression pattern to certain tissues compared to pancRNA-lacking genes, 2) expression of pancRNAs was significantly correlated with the enrichment of active chromatin marks, H3K4 trimethylation and H3K27 acetylation, at the promoter regions of the partner genes, 3) H3K4me1 marked the pancRNA-partnered genes regardless of their expression level, and 4) C- or G-skewed motifs were exclusively overrepresented between−200 and−1 bp relative to the transcription start sites of the pancRNA-partnered genes. More importantly, the comparative transcriptome analysis among five different mammalian species using a total of 25 counterpart tissues showed that the overall pancRNA expression profile exhibited extremely high species-specificity compared to that of total mRNA, suggesting that interspecies difference in pancRNA repertoires might lead to the diversification of mRNA expression profiles.

**Conclusions:**

The present study raises the interesting possibility that the gain and/or loss of gene-activation-associated pancRNA repertoires, caused by formation or disruption of the genomic GC-skewed structure in the course of evolution, finely shape the tissue-specific pattern of gene expression according to a given species.

**Electronic supplementary material:**

The online version of this article (doi:10.1186/s12864-017-3662-1) contains supplementary material, which is available to authorized users.

## Background

Comparative genomics enables one to identify highly conserved genomic sequences over the course of evolution. The majority of such sequences, frequently located within protein-coding regions accounting for a few percent of the mammalian genome, have been thoroughly studied, resulting in the identification of functional protein domains that are important for the living organisms [1]. Similarly, it has been shown that highly conserved genomic sequences are also located in a set of regulatory sequences that activate or repress gene transcritions in a wide range of animals [2, 3]. However, it remains largely unknown how protein structure and gene expression pattern is differentiated according to a given species.

At present, phenotypic diversity is thought to be more likely to result from the changes in transcriptional regulation than from those in protein function. Over the course of evolution, protein-coding sequences are better conserved among species in comparison to the sequences of non-coding regions [4]. Changes in protein-coding regions can alter amino acid sequences, frequently leading to alteration of the functional properties of proteins. Since such mutated proteins are frequently deleterious to a wide range of cell types, the corresponding mutations, if any, are somehow removed by negative selection in a population. In contrast, changes in the bulk non-coding genomic regions are much less harmful to the organisms except for some ultraconserved regions that can not tolerate changes of their sequences [5]. Unlike protein-coding regions, gene regulatory regions, such as cell-type specific enhancers, tend to show much more diversified sequences according to the species. This is presumably because mutations in these gene regulatory regions are deleterious to only a limited number of cells, but not to all cell types. In fact, several gene regulatory elements responsible for the expression of phenotypic differences among species have been identified [6–11]. For example, human-specific loss of the enhancer at the promoter regions of the androgen receptor gene is implicated in the loss of sensory vibrissae and penile spines [12]. Taken together, it is likely that alterations in the DNA sequences at cell-type-specific regulatory elements allow evolutionary changes to adapt a given species according to its environment.

Recent transcriptome analyses have found that the non-coding genomic regions provide templates for generating thousands of long non-coding RNAs (lncRNAs): transcription occurs at more than 60% of the mammalian genomic DNA [13, 14]. Accumulating evidence shows that lncRNAs play a key role in transcriptional or posttranscriptional regulation in a genome-wide fashion [15–18]. For example, *HOTAIR* induces repressive chromatin formation with polycomb repressive complex 2 and Lysine specific demethylase 1, leading to decreases in the expression level of hundreds of protein-coding genes [19, 20].

In addition to the example of functional lncRNAs, we have shown that a set of lncRNAs transcribed from bidirectional promoters, promoter-associated non-coding RNAs (pancRNAs), could activate the expression of the partner genes through sequence-specific alterations in the epigenetic status at their promoter regions [21–23]. For instance, *pancVim*, which is transcribed from the promoter region of the vimentin gene (*Vim*), could induce sequence-specific DNA demethylation, demethylation of lysine 9 of histone 3 (H3K9) and methylation of lysine 4 of histone 3 (H3K4), leading to the activation of *Vim* expression in a cell-type-specific manner in rat PC12 cells [22]. *Khps1*, a pancRNA for sphingosine kinase 1 (*Sphk1*), could also induce the formation of active chromatin structure in a tissue-specific manner [21]. Later on, other groups confirmed the occurrence of similar phenomena in the human *VIM* [24] and *SPHK1* [25] loci. Temporal regulation of the expression of pancRNAs also plays an essential role in mammalian development. For example, *pancIl17d*, a pancRNA for interleukin 17d, is essential for embryonic survival and for maintaining stem cell pluripotency by mediating sequence-specific DNA demethylation together with ten-eleven translocation 3 and poly (ADP-ribose) polymerase [23]. Furthermore, several gene-activating pancRNAs play essential roles in terminal differentiation processes of rat PC12 cells [26]. Thus, spatiotemporal transcriptional regulation mediated by pancRNA seems to function throughout life.

Widely occurring but context-dependent expressions of pancRNAs are observed not only in rodent tissues but also in primate tissues, raising the possibility that the pancRNA-mediated regulatory mechanism is utilized in common across mammalian species [27]. In order to examine this possibility, we have started to perform comparative transcriptome analysis with directional RNA sequencing (RNA-seq) data of five tissues (cerebral cortex, cerebellum, heart, kidney and liver) form five species (chimpanzee, macaque, marmoset, mouse and rat).

## Methods

### Tissue preparation

C57BL/6 mice (*Mus musculus*; Japan SLC) were kept under a lighting regime of 14 h illumination and 10 h darkness (lights on between 05:00 and 19:00) and were allowed free access to food and water. Tissue samples for directional RNA-seq preparation from C57BL/6 mice (16 weeks of age; male) were collected and immediately frozen in liquid nitrogen and stored at−80 °C until use. Thanks to the Great Ape Information Network (GAIN) and Kumamoto Sanctuary, Wildlife Research Center, Kyoto University, the Brodmann area 10 and the heart were collected from a chimpanzee (*Pan troglodytes*; 28-year-old female) and the cerebral cortex and the cerebellum were collected from a macaque (*Macaca mulatta*; about 1-year-old male). The total RNAs were isolated from the mouse heart, the macaque cerebral cortex and cerebellum, and the chimpanzee cerebral cortex and heart.

### Directional RNA sequencing

Directional RNA-seq samples were prepared according to a slight modification of the protocol provided by Illumina. Briefly, cDNA libraries were prepared starting from 5 μg of total RNA from one individual as follows. We previously showed that poly A+ pancRNA overexpression upregulates the partner mRNA expression [23, 26, 27]. First, total RNA was selected twice with Sera-Mag Magnetic Oligo dT Beads (Thermo Scientific) to isolate polyA+ RNA. The fraction of rRNA was found to be less than 2% in each polyA+ RNA sample by using a Total RNA Pico Bioanalyzer chip (Agilent Technologies). polyA+ RNA was fragmented by heating at 94 °C for 3 min in fragmentation buffer (Affymetrix), followed by ethanol precipitation. Fragmented RNA was decapped with Tobacco Acid Pyrophosphatase (Nippongene), followed by extraction with PCI and ethanol precipitation. Fragmented and decapped RNA was 3′-dephosphorylated using Antarctic phosphatase (New England Biolabs). The RNA was 5′-phosphorylated using T4 polynucleotide kinase (New England Biolabs). The modified RNA was cleaned up with an RNeasy MinElute kit (QIAGEN). The RNA was ligated to 1 × v1.5 sRNA 3′ adaptor (Illumina) with T4 RNA ligase 2, truncated K277Q (New England Biolabs) at 4 °C overnight. This RNA was ligated to SRA 5′ adaptor (Illumina) with T4 RNA ligase (Illumina) at 20 °C for 1 h. cDNA was synthesized with specific RT primer and the SuperScriptIII First-Strand Synthesis System (Life Technologies). After the amplification of cDNA libraries, the PCR product was purified twice with AMPure XP (Beckman Coulter) to generate a library and analyzed on a DNA1000 Bioanalyzer chip (Agilent Technologies) for precise quantification of molarity. After confirmation of the high quality of the cDNA library samples, Illumina HiSeq 2000 was used to perform single-end sequencing with the small RNA sequencing primer (Illumina) according to the manufacturer’s instructions. Our RNA-seq data have been deposited in the DDBJ Sequence Read Archive (DRA000861, DRA003227, DRA003228).

### Directional RNA-seq data processing

The directional RNA-seq dataset used in this study consists of 12 new and 63 publicly available samples (Additional file 1: Table S1) [27–31]. In order to process all directional RNA-seq data in the same way, only reads corresponding to the upstream side of the original transcript were extracted from paired-end reads and treated as single-end reads. Reads of all directional RNA-seq data were assessed with the FASTX toolkit (http://hannonlab.cshl.edu/fastx_toolkit/index.html) to eliminate low quality (quality score less than 20) nucleotides and the adaptor sequence from the 3′-end of reads, followed by removal of short (less than 20 nt) reads. Preprocessed reads were mapped to the reference genome of the corresponding species using TopHat v2.0.8 [32] and Bowtie v1.0.0 [33]. The reference genome sequences of chimpanzee (panTro4), macaque (rheMac3), marmoset (calJac3), mouse (mm10) and rat (rn5) were retrieved from the UCSC Genome Browser database [34]. In order to verify the strandedness of directional RNA-seq data, whether the strandedness of reads mapped to the known protein-coding regions was concordant with the strandedness of reference genes was verified using RSeQC v2.3.6 [35].

### Normalization and estimation of mRNA and pancRNA expression levels

For quantification of mRNA expression, the reads uniquely mapped to each protein-coding gene were counted using HTSeq v0.6.0 [36]. The protein-coding gene models of the genome of each species were obtained from the Ensembl Gene track in the UCSC Genome Browser database. Because there is no Ensembl Gene track available from the UCSC Genome Annotation database for the rheMac3 genome, the positions of protein-coding gene models of the rheMac2 genome were converted to the rheMac3 genome assembly by using the UCSC LiftOver tool [34] because there are no one-to-one ortholog data between rheMac2 and other species in the Ensemble Compara database utilized for cross-species transcriptome analysis. In order to quantify pancRNA expression, the reads uniquely mapped to the antisense sequences of the promoter regions (−2000 to−1 bp from the transcription start sites (TSSs)) of protein-coding genes were counted using HTSeq v0.6.0. When a promoter region overlapped with another gene or another promoter region, or was close to another promoter region (<500 bp), the most distal promoter was used in our analysis after removing the distal promoters which overlapped with another gene or promoter region to avoid contamination of the pancRNA pool by protein-coding genes. In order to calculate the gene expression levels, the read counts for the data of each species were normalized by the DEGES-based normalization method implemented in TCC [37].

In this study, the definition of a pancRNA-partnered gene is a protein-coding gene whose expression level is positively correlated with those of the corresponding pancRNA across the five tissues (Pearson correlation coefficient > 0.7). The definition of a pancRNA-lacking gene is a protein-coding genes whose expression level is not positively correlated with those of the corresponding pancRNA across the five tissues (Pearson correlation coefficient < 0.4). The correlation coefficients were calculated using the cor function in R (http://www.R-project.org/).

### Quantification of the tissue specificity of the gene expression pattern

The tissue specificity of the gene expression pattern was quantified with tissue-specificity index (TSI) [38], which varies between zero and one. Values close to one represent high tissue specificity. The Steel-Dwass method was used for comparisons among four groups. Graphical representations were done with the ggplot2 package (http://ggplot2.org).

### Visualization of mouse ChIP-seq data

Ngsplot v.2.47. software [39] was used to visualize the enrichment pattern of each histone modification. Bam-formatted data of chromatin immunoprecipitation together with DNA sequencing (ChIP-seq) used in this study were obtained from Mouse ENCODE Downloads in the UCSC Genome Browser database (Additional file 2: Table S2).

### *De novo* motif discovering

For discovering continuous motifs, the−200 to−1 bp sequences (relative to the TSS) of each group of genes were examined using MEME v.4.10.0 [40]. In the analysis with MEME, we set the -mod option to zoops and the -nmotifs option to 4. We calculated the average observed frequency of sequences showing 70% or more identity to each motif in genomic regions around TSSs (−2,000 to +2,000 bp relative to the TSS) with a sliding window of width 50 bp using the matchPWM program in the Biostrings package v.2.30.1 (http://www.bioconductor.org/packages/2.11/bioc/html/Biostrings.html).

### Quantification of the sequence conservation

For quantification of the sequence conservation, the phastCons score for multiple alignments of 45 Euarchontoglires’ genomes with mouse available from the UCSC Genome Browser database was utilized. In this analysis, the promoter region is defined as the region from−2,000 to−1 bp relative to the TSS. In order to quantify the sequence conservation of protein-coding regions, the average phastCons score for all exonic regions coding for amino acids was calculated. The Steel-Dwass method was used for comparisons among four groups (coding sequence regions (CDS), promoter regions of total genes, those of pancRNA-partnered genes, and those of pancRNA-lacking genes). Graphical representations were done with the ggplot2 package (http://ggplot2.org).

### Comparative transcriptome analysis

The list of one-to-one orthologous genes for each pair of the five species was retrieved from the Ensembl Compara database, release 78 [41]. Hierarchical clustering of sequenced samples based on the gene expression level was carried out using the hclust function in R (http://www.R-project.org/). The distance between samples was computed as 1 - ρ, where ρ is the Spearman correlation coefficient. Symmetrical heatmaps of Spearman correlations from the mean of the average gene expression profiles of replicates were drawn using the heatmap.2 function in the gplots package (http://cran.r-project.org/web/packages/gplots/index.html). For the inter-species comparison of the pancRNA expression profile, the expression levels of transcripts from the promoter regions whose orthologous regions encode the pancRNAs in any species were calculated. In this heatmap, dendrograms were drawn based on hierarchical clustering of pairwise Spearman correlations. In this study, a species-specific pancRNA-partnered gene was defined as a pancRNA-partnered gene in one species of which all orthologous genes in the other species are pancRNA-lacking genes.

## Results

### Identification of thousands of pancRNA-partnered genes in five different mammalian species

In order to understand the pancRNA expression profile in mammals, we used directional RNA-seq data of five types of tissues (cerebral cortex, cerebellum, heart, kidney and liver) from five species (chimpanzee, macaque, marmoset, mouse and rat; Additional file 1: Table S1; see the Methods section). This transcriptome dataset from 25 samples consists of a total of approximately 6 billion directional RNA-seq reads. We mapped these reads to the relevant genomes (for example, data for chimpanzee to the panTro4 genome). We next verified the strandedness of these RNA-seq data and found that, on average, about 97.5% of the reads from each sample were mapped to the correct strand of the known protein-coding genes (Additional file 1: Table S1). RNA-seq data utilized in this study showed robust reproducibility of mRNA expression levels among replicates of each tissue samples from each species (Spearman correlation coefficient, *ρ* > 0.9; Additional file 3: Figure S1).

To identify the pancRNA-partnered genes in each species, we calculated the Pearson correlation coefficients between the pancRNA candidate and the cognate mRNA expression levels in the five tissues. In this study, we defined pancRNA-partnered genes as the protein-coding genes whose expression level is positively correlated with those of the corresponding pancRNA across the five tissues (Pearson correlation coefficient > 0.7). While 157, 83, 74, 102 and 75 pancRNA-mRNA pairs showed negative correlation of their expression levels, 2013, 1293, 1588, 3229 and 1835 pancRNA-mRNA pairs showed positive correlation of their expression levels in chimpanzee, macaque, marmoset, mouse, and rat, respectively (Table 1), indicating that the majority of pancRNA-mRNA pairs show positive correlation between their expression levels This is consistent with previous reports [23, 27, 42, 43], supporting the validity of our transcriptome analysis in this study. In this way, we identified thousands of pancRNA-partnered genes in each species (Table 1). This result suggests that the pancRNA-mediated transcriptional activation mechanism exists in common across the five mammalian species. The number of pancRNA-partnered genes varied among the five species, possibly because of the difference in sequencing depth of RNA-seq and in enrichment of gene annotations among the five species. For example, in the mouse transcriptome analysis, we identified 3.2 thousand pancRNA-partnered genes with about 2.1 billion mapped reads. On the other hand, in the marmoset analysis, we identified only 1.6 thousand pancRNA-partnered genes with about 232 million mapped reads.

**Table 1:** The pancRNA-partnered genes in the five species

Species	Total protein-coding genes^a^	Positive correlation^b^ (pancRNA-partnered genes)	(%)^c^	Negative correlation^d^	(%)^e^
Chimp	15036	2013	13.4%	157	1.0%
Macaque	11745	1293	11.0%	83	0.7%
Marmoset	15125	1588	10.5%	74	0.5%
Mouse	17193	3229	18.8%	102	0.6%
Rat	18715	1835	9.8%	75	0.4%

^a^Total protein-coding genes excluding genes containing parts of other genes within their promoters

^b^The protein-coding genes whose expression level is positively correlated with those of the corresponding pancRNA (*P* < 0.05)

^c^The percentage of the protein-coding genes whose expression level is positively correlated with those of the corresponding pancRNA in total protein-coding genes

^d^The protein-coding genes whose expression level is negatively correlated with those of the corresponding pancRNA (*P* < 0.05)

^e^The percentage of the protein-coding genes whose expression level is negatively correlated with those of the corresponding pancRNA in total protein-coding genes

### pancRNA-partnered genes show highly tissue-specific expression patterns

**Fig. 1:**
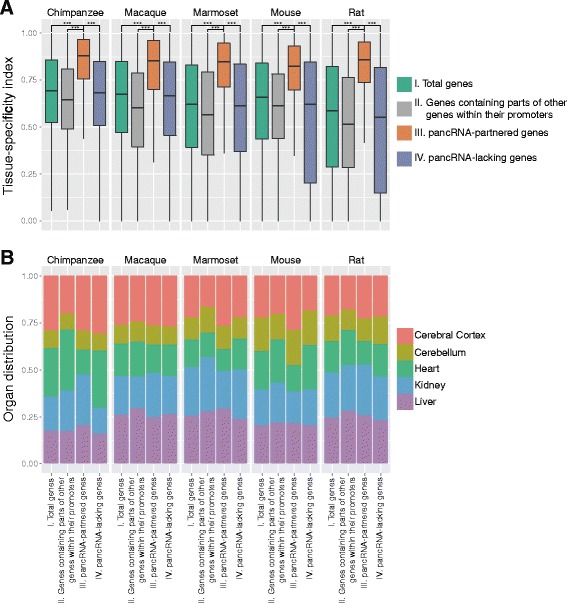
Tissue-specificity of the pancRNA-partnered gene expression. **a** Tissue-specificity index of total protein-coding genes, of genes containing parts of other genes within their promoters, of pancRNA-partnered genes, and of pancRNA-lacking genes. *** *P* <0.001; Error bars indicate the first and third quartiles. **b** Distribution of the tissue in which the expression level of protein-coding genes is the highest, for total protein-coding genes, for genes containing parts of other genes within their promoters, for pancRNA-partnered genes, and for pancRNA-lacking genes

To examine whether the pancRNA-partnered genes tend to be tissue-specifically expressed using the genome-wide approach, we evaluated the tissue-specificity of gene expression by calculating the TSI [38] as described in Methods. We compared the TSI of four subclasses of protein-coding genes among the five species: I) total protein-coding genes, II) protein-coding genes containing parts of other protein-coding genes within their promoter regions, III) pancRNA-partnered genes and IV) pancRNA-lacking genes. The TSI for class III was significantly higher than that for other classes (Fig. 1a). In particular, it is interesting to note that the TSI for class II was significantly lower than that for class III. This suggests that bidirectional promoter activity itself does not increase the TSI; rather, the expression of pancRNA might restrict the partner gene expression to only limited tissues. In fact, the tissue-specificity of pancRNA itself is also high, as is that of pancRNA-partnered genes (Additional file 4: Figure S2). To further investigate the characteristics of the pancRNA-partnered gene expression pattern, we next examined if the group of the pancRNA-partnered genes were expressed preferentially in a tissue, and found no tissue bias (Fig. 1b). The fact that this tendency was commonly shared in the five species (Fig. 1b) implies that various tissues have comparable capacity to express pancRNA repertoires for the partner gene expression.

### H3K4me1 enrichment marks the template regions of pancRNAs regardless of their expression

**Fig. 2:**
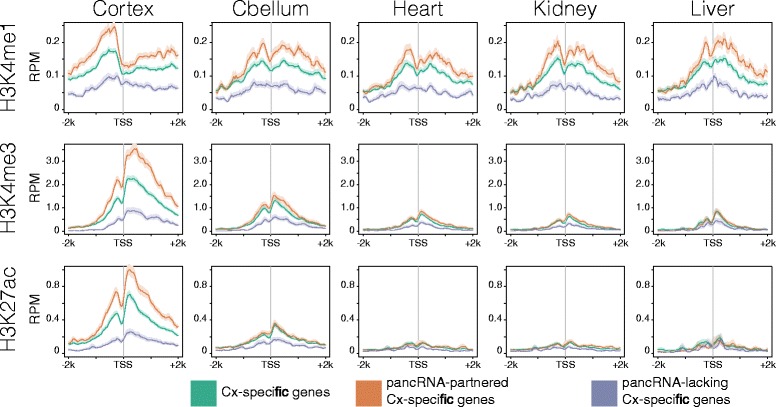
Histone modifications across the regions around TSSs of cerebral cortex-specific genes. RPM (Read count per million mapped reads) derived from ChIP-seq data (H3K4me1, H3K4me3, and H3K27ac) of mouse cerebral cortex (Cortex), cerebellum (Cbellum), heart, kidney, and liver across the regions around TSSs (−2,000 bp to +2,000 bp relative to TSS). In this analysis, cerebral cortex-specific genes, pancRNA-partnered cerebral cortex-specific genes, and pancRNA-lacking cerebral cortex-specific genes were utilized (TSI > 0.9). The standard error of the mean across the regions is shown as semi-transparent shading around the mean curve

We next investigated whether the expression of pancRNAs was associated with the establishment of the histone modification pattern. Using ChIP-seq data in the mouse ENCODE database (Additional file 2: Table S2) [44], we examined the enrichment of the histone modifications at the regions around TSSs of protein-coding genes, pancRNA-partnered genes and pancRNA-lacking genes that represent the tissue-specific expression pattern (TSI > 0.9). Because pancRNAs have been shown to be involved in the active chromatin modification, we focused on mono-methylated H3K4 (H3K4me1), tri-methylated H3K4 (H3K4me3) and acetylated lysine 27 of histone H3 (H3K27ac). At the regions around TSSs of the protein-coding genes, both H3K4me3 and H3K27ac were frequently observed in the tissue where the genes show the maximum expression level in comparison to the other four tissues (Fig. 2, Additional file 5: Figure S3). Intriguingly, in the tissue where the genes show the maximum expression level, H3K4me3 and H3K27ac were more frequently observed at the regions around TSSs of the pancRNA-partnered genes than at those of protein-coding genes and pancRNA-lacking genes (Fig. 2, Additional file 5: Figure S3). These results indicate that the expression of pancRNA is strongly associated with the enrichment of the active chromatin modification, and suggest that the establishment of H3K4me3 and H3K27ac marks might play a key role in triggering expression of pancRNA-partnered genes in a tissue-specific manner. However, considering the expression levels of pancRNA-partnered tissue-specific genes, we cannot completely exclude the possibility that the enrichment of active chromatin marks at these promoters might simply be a sign that pancRNA-partnered genes are more highly expressed than other protein-coding genes (Additional file 6: Figure S4). At the regions around TSSs of pancRNA-partnered genes, H3K4me1 was more frequently observed regardless of the tissue than at the TSSs of protein-coding genes and pancRNA-lacking genes (Fig. 2, Additional file 5: Figure S3). This tendency of H3K4me1 enrichment in the promoter regions of pancRNA-partnered genes raised the possibility that the promoter regions of pancRNA-partnered genes were epigenetically marked as a result of particular sequence features that had enabled them to acquire pancRNA-coding regions in the genomic DNA.

### Genomic characteristics of the promoter regions of pancRNA-partnered genes

**Fig. 3:**
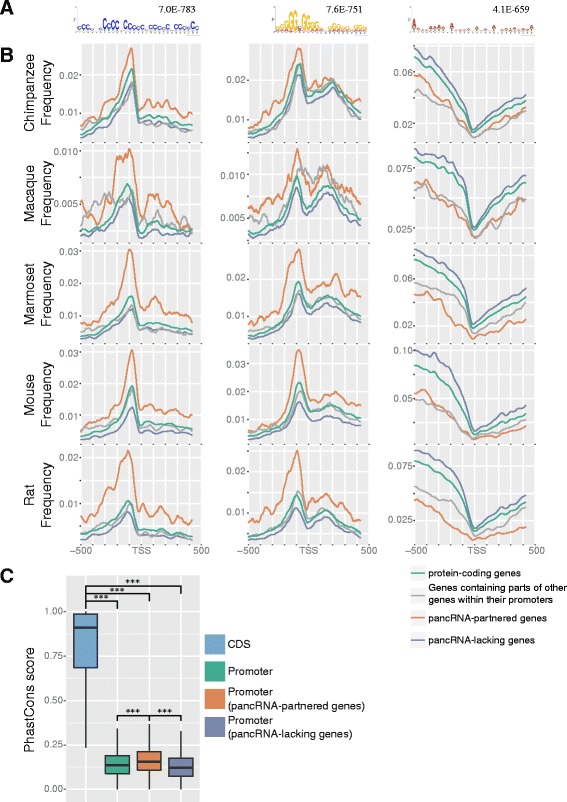
Genomic features of promoter sequences of pancRNA-partnered genes. **a**. The DNA motifs enriched in the immediately upstream regions of TSSs of pancRNA-partnered genes in the mouse genome (−200 bp to −1 bp relative to the TSS). The E-value of each motif is shown. **b**. Observed frequency of each DNA motif shown in panel A in the regions around TSSs (−500 bp to +500 bp relative to the TSSs) of each group of genes (total protein-coding genes, genes containing parts of other genes within their promoters, pancRNA-partnered genes, and pancRNA-lacking genes) in the five species’ genomes. **c**. Sequence conservation (phastCons score) for coding sequence regions (CDS), promoter regions of total genes, those of pancRNA-partnered genes, and those of pancRNA-lacking genes. *** *P* <0.001; Error bars indicate the first and third quartiles

It is possible that the epigenetic characteristics of the pancRNA-partnered genes are further characterized by enrichment of some specific DNA sequences. We and another group previously reported that C-rich or G-rich sequences exist biasedly around the TSS at the immediate upstream regions of the TSSs of pancRNA-partnered genes [23, 27]. In agreement with these reports, we found that the enrichment of CpG islands in the promoter regions of pancRNA-partnered genes (retrieved from the UCSC Genome Browser database) was higher than that in either the category of all protein-coding genes or the category of pancRNA-lacking genes in the five species (Additional file 7: Table S3), and we identified C- and G-skewed motifs, which showed biased enrichment of cytosines and guanines, respectively, in the immediate upstream regions of TSSs (−200 to−1 bp) of pancRNA-partnered genes in the genome of all five species examined here (Fig. 3a, Additional file 8: Figure S5). Analysis of the distribution of these motifs at the regions around TSSs confirmed that the C- and G-skewed motifs were more frequently observed in the immediate upstream regions of TSSs of pancRNA-partnered genes than in those of pancRNA-lacking genes in all of the five species (Fig. 3b). Of these C- and/or G-skewed motif-bearing immediate upstream regions of TSSs of pancRNA-partnered genes, about 16.4% harbored both of these two motifs in all five species (Additional file 9: Table S4). Thus, the presence of either C- or G-skewed motifs in the immediate upstream regions of TSSs is a genomic feature of pancRNA-partnered genes.

On the assumption that the C- or G-skewed motifs are important for pancRNA transcription, such a motif should have been conserved once acquired. In order to evaluate the degree of the sequence conservation, we utilized the phastCons score for multiple alignments of 45 Euarchontoglires genomes [45]. It is logical that protein-coding regions were much more strongly conserved than promoter regions (Fig. 3c), since changes in promoter regions are less deleterious to a wide range of cell types than those in protein-coding regions. Next, we examined the phastCons scores of two subclasses of promoter regions: promoter regions of pancRNA-partnered genes and those of pancRNA-lacking genes. Interestingly, we found that the promoter regions of pancRNA-partnered genes exhibited a higher level of sequence conservation than those of pancRNA-lacking genes (Fig. 3c). This difference in sequence conservation was small but significant (*P* < 0.001), raising the possibility that negative selection acted to conserve the promoter sequence once pancRNAs started to participate in transcriptional regulations in the course of evolution.

### The expression profile of pancRNA exhibits extremely high species-specificity

**Fig. 4:**
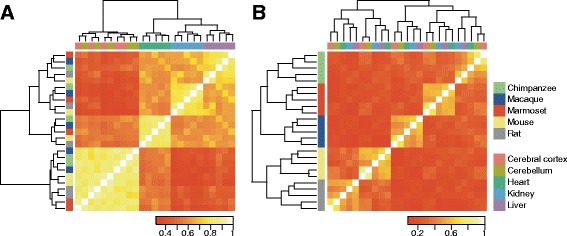
Diversity of pancRNA expression profile of the five tissues in the five species. Hierarchical clustering and symmetrical heat map of Spearman correlations of mRNA (**a**) and pancRNA (**b**) expression profiles. Samples are colored according to the tissues and the species

In order to assess the degree to which mRNA and pancRNA expression profiles are diversified among mammalian species, we calculated the correlation coefficients of the mRNA and pancRNA expression levels across all pairs of samples. When samples were clustered on the basis of mRNA expression profile, they were segregated according to tissue type (Fig. 4a). Notably, on the other hand, when samples were clustered on the basis of pancRNA expression profile, they were segregated by individual species (Fig. 4b). Close inspection of the hierarchical clustering data revealed the values for the cerebral cortex and cerebellum, for example, were located next to each other in each species (Additional file 10: Figure S6), and therefore, the segregation of the pancRNA expression profile according to species did not indicate low tissue diversity of the pancRNA expression profile, but rather showed the extremely high species diversity of the pancRNA expression profile. When the expression profile of conserved pancRNAs was extracted for clustering analysis, the samples were confirmed to be segregated according to tissue type, as is the case for the clustering data of the mRNA expression profile (Additional file 11: Figure S7), meaning that the majority of the pancRNAs are not well conserved over species in terms of their expression pattern. [23, 27, 42, 43] Species-specific-pancRNA-partnered genes exhibit similar features to the bulk of pancRNA-partnered genes.

Considering the role of pancRNAs in transcriptional regulation and the high diversity of pancRNA expression profiles among mammals, we hypothesized that the species-specific gain and loss of pancRNA expression ability has diversified the mRNA expression profile according to the mammalian species. Of the pancRNA-partnered genes in the five species, we identified species-specific-pancRNA-partnered genes by the comparison of their orthologous gene expression profiles. The number of genes that had a partner pancRNA in only one species was 103, 79, 67, 220 and 55 in chimpanzee, macaque, marmoset, mouse and rat, respectively (Table 2, Additional file 12: Table S5). By contrast, the number of genes that lacked pancRNA in a certain species was 6, 10, 11, 5, and 18 in chimpanzee, macaque, marmoset, mouse and rat, respectively, suggesting that a gain of pancRNAs might have occurred more frequently than a loss over the course of evolution. It should be noted that the variation in the number of species-specific pancRNA-partnered genes might depend on the depth of transcriptome data (Additional file 1: Table S1).

**Table 2:** The number of species-specific pancRNA-partnered genes in the five species

Species	pancRNA-partnered genes^a^(One-to-one orthologs)	Species-specific pancRNA-partnered genes	(%)^b^
Chimp	1427	103	7.2%
Macaque	890	79	8.9%
Marmoset	1134	67	5.9%
Mouse	2219	220	9.9%
Rat	1283	55	4.3%

^a^The singleton orthologous pancRNA-partnered genes present in all five species

^b^ The percentage of the species-specific pancRNA-partnered genes in the pancRNA-partnered genes

**Fig. 5:**
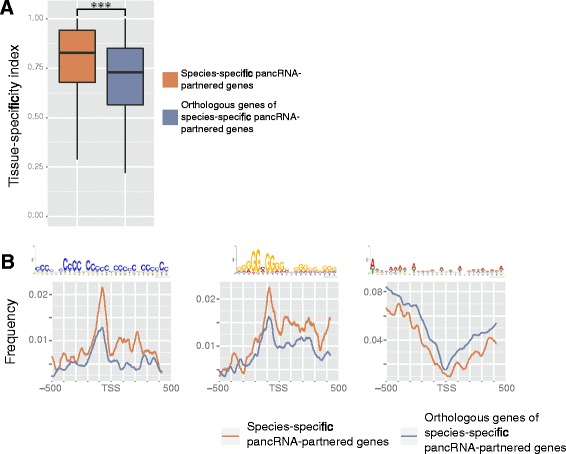
Expression pattern and genomic features of species-specific pancRNA-partnered genes. **a**. Tissue-specificity index of species-specific pancRNA-partnered genes and their orthologous genes in the four other species. *** *P* <0.001; The error bars indicate the first and third quartiles. **b**. Observed frequency of each DNA motif shown in Fig. 3a at the regions around TSSs (−500 bp to +500 bp relative to the TSSs) of each group of genes (any species-specific pancRNA-partnered genes and their orthologous genes in the other four species)

In order to confirm that the species-specific-pancRNA-partnered genes show tissue-specific expression patterns, we calculated TSIs of the expression of species-specific-pancRNA-partnered genes and those of their pancRNA-lacking orthologous genes in the other four species. We found that the average TSI of species-specific pancRNA-partnered genes’ expression was significantly higher than that of their orthologous genes’ expression (*P* < 0.001; Fig. 5a). In the regions between−200 and−1 bp relative to TSSs of genes that had a partner pancRNA only in one species, these C- and G-skewed motifs were observed more frequently than in those of their orthologous genes (Fig. 5b). This agrees well with the discovery of the C- and G-skewed motifs in the promoters of pancRNA-partnered genes (Fig. 3). Therefore, species-specific-pancRNA-partnered genes share several common genomic features with the bulk pancRNA-partnered genes.

## Discussion

Using directional RNA-seq data of five types of tissues from five mammalian species, we identified thousands of pancRNAs in every species. We found several common features of the pancRNA-partnered genes: 1) pancRNA-partnered genes showed highly tissue-specific patterns of expression (Fig. 1a), 2) expression of pancRNAs was significantly correlated with the enrichment of active chromatin marks at the promoter regions of the partner genes (Fig. 2), 3) H3K4me1 marked the pancRNA-partnered genes regardless of their expression level (Fig. 2), and 4) C- or G-skewed motifs were preferentially observed between −200 and −1 bp relative to TSSs of the pancRNA-partnered genes (Fig. 3b). These results suggest that pancRNA-partnered genes are genetically and epigenetically regulated for their activation regardless of the species.

Surprisingly, the comparative transcriptome analysis showed that the expression profile of pancRNA exhibited much higher species-specificity than that of mRNA (Fig. 4), suggesting that a significant number of pancRNAs were differentially expressed among species for enhancing expression of a set of partner genes in a species-specific manner. Comparison of species-specific pancRNA-partnered genes with their orthologous pancRNA-lacking genes confirmed that species-specific pancRNA-partnered genes showed more tissue-limited patterns of expression, and that C- or G-skewed motifs were enriched at the promoter regions of species-specific pancRNA-partnered genes (Fig. 5). Thus, we believe that the evolutionary acquisition of gene-activating pancRNA, caused by the asymmetrical genomic structure due to an increase in C- or G-skew at the bidirectional promoters, enhances the tissue-specificity of the partnered gene expression, as further discussed below.

### General existence of gene activation mechanism mediated by pancRNAs in mammals

Several pancRNAs have been shown to act to enhance the expression level of the corresponding mRNAs *in cis* [21–23, 27]. We found that the expression of pancRNA-partnered genes was strongly associated with H3K4me3 and H3K27ac enrichment (Fig. 2). In accordance with this general tendency, we previously showed that knockdown and over-expression of pancRNA for *Vim* induced a decrease and increase of H3K4 methylation, respectively, at the promoter region of the corresponding gene [22]. Although we could not examine the DNA methylation status at the genome-wide level because of the lack of an appropriate dataset, pancRNA-partnered genes were associated with all of the active histone marks examined. Therefore, the present data are in agreement with our proposition that thousands of pancRNAs facultatively induce the recruitment of active epigenetic marks, leading to the transcriptional activation of the partner genes.

Intriguingly, we found that H3K4me1 was more enriched at the promoter regions of pancRNA-partnered genes than at those of pancRNA-lacking genes and protein-coding genes. In other words, the template regions of pancRNA were characterized by constitutive H3K4me1 enrichment regardless of their transcriptional activity, unlike H3K4me3 and H3K27ac (Fig. 2). It is known that active and poised promoters could be distinguished by the presence or absence of H3K27ac, respectively, and that H3K4me1 is enriched at enhancer regions for the target genes regardless of their expression [46, 47]. Taking this general information into account, the uniform enrichment of H3K4me1 at the promoter regions of pancRNA-partnered genes suggests that pancRNA-expressing promoter regions could act not only as typical promoters for triggering the transcription of mRNAs but also as enhancers for further increasing the expression level of the partner genes.

### Interspecies diversity of the pancRNA expression profile for the evolutionary fine tuning of tissue-specific gene expression

It is known that transcriptional regulation of regulatory genes, such as transcription factors, tends to be well conserved among species via conservation of the responsible DNA sequences [48]. Some lncRNAs also show high sequence conservation. For example, *Tuna*, which is involved in the transcriptional regulation of thousands of genes, is the conserved lncRNA indispensable for maintenance of pluripotency and neural differentiation of mouse embryonic stem cells [49]. In contrast, high diversity in the promoter sequences suggests that the promoter regions are tolerant to changes at the genomic DNA-level, ultimately leading to the gain or loss of pancRNA-partnered genes. It is interesting to note that non-conservation of ncRNAs does not mean that such ncRNAs are not functional. Since the gain of pancRNA has occurred more frequently than the loss (Table 2, Additional file 12: Table S5), the species-specific changes of pancRNA expression might not impose natural selection over the course of evolution; rather, these changes might have been utilized for the diversification of transcriptional regulation of their cognate genes. Our comparative transcriptome analysis showed that the diversity of the pancRNA expression profile was higher than that of the mRNA expression profile (Fig. 4). The validity of this result is supported by recent reports showing that expression patterns of lncRNAs have evolved at a more rapid rate than those of mRNAs [50]. Here, we propose the necessity of focusing on the nonconserved ncRNAs, such as pancRNA, to understand the evolutionary diversification of the transcriptome.

### Gain of pancRNAs, caused by formation of the genomic GC-skewed structure, finely specifies the cognate gene expression pattern in tissues

We propose that, once pancRNAs have participated in transcriptional regulations of the partner genes, negative selection has acted to maintain the pancRNA expression. This proposition is supported by our finding here that the promoter regions of pancRNA-partnered genes exhibit a higher level of sequence conservation than those of pancRNA-lacking genes (Fig. 3c). We also showed the higher enrichment of C- or G-skewed motifs in the promoter regions of pancRNA-partnered genes than in those of pancRNA-lacking genes. Taken together, these findings support an evolutionary scenario in which increases in the frequencies of C- or G-skewed motifs in promoter regions contribute to pancRNA expression, and thereafter, such sequences becomes conserved at the promoter regions.

Species-specific pancRNA-partnered genes show DNA-level and transcription-level characteristics that are similar to the bulk of pancRNA-partnered genes (Fig. 5). The highly organized expression patterns of species-specific pancRNA-partnered genes suggests that a certain species might gain pancRNA expression for the adaptation of tissue function through the cognate gene regulation. The frequent occurrence of C- and G-skewed motifs between−200 and−1 bp relative to TSSs of pancRNA-partnered genes raises the possibility that the expression of pancRNAs has been acquired at various gene loci in a species-dependent manner partly due to the increase of the C or G frequency at the immediate upstream regions of TSSs. Considering the bidirectional promoter activity of regions with high GC content, such as CpG islands [51, 52], we propose that the appearance of bidirectional promoter activity at the GC-rich promoter regions plays an important role in the process of pancRNA acquisition for the cognate gene to be more tissue-specifically expressed. We do not yet know whether occasional pancRNA expression at the CpG island-type promoters has been fixed later at the DNA-level. Nonetheless, it would be interesting to test the idea that ncRNA-mediated epigenetic changes are the driver for the genetic alteration to adapt gene-expression patterns according to the mammalian species.

## Conclusions

The present study raises the interesting possibility that the changes of gene-activation-associated pancRNA repertoires, partly caused by formation of a genomic GC-skewed structure, finely shape tissue-specific patterns of gene expression according to a given species. pancRNA should constitute a new layer of species-dependent gene activation mechanism for the generation and adaptation of a species.

## Additional files

Additional file 1: Table S1. Directional RNA-seq used in this study. (XLS 63 kb)

Additional file 2: Table S2. Mouse ENCODE ChIP-seq data used in this study. (XLS 28 kb)

Additional file 3: Figure S1. Hierarchical clustering of directional RNA-seq data. Dendrogram represents average linkage hierarchical clustering of directional RNA-seq data based on the mRNA expression profiles in each of the five species. The distance between data was computed as 1 − *ρ*, where *ρ* is the Spearman correlation coefficient. Note that the gene expression profiles of 16-week-old mice (mouse cerebral cortex sample #1-4; home-made RNA-seq data) and 8-week-old mice (mouse cerebral cortex sample #5-6; RNA-seq data from the mouse ENCODE project) are quite similar to each other. (PDF 60 kb)

Additional file 4: Figure S2. Tissue-specificity index of total protein-coding genes, of pancRNA-partnered genes, and of pancRNAs. *** *P* <0.001; Error bars indicate the first and third quartiles. (PDF 66 kb)

Additional file 5: Figure S3. Histone modification across the regions around TSSs of each tissue-specific gene. RPM (Read count per million mapped reads) derived from ChIP-seq data (H3K4me1, H3K4me3, and H3K27ac) of mouse cerebral cortex (Cortex), cerebellum (Cbellum), heart, kidney, and liver across the regions around TSSs (−2,000 bp to +2,000 bp relative to TSS). In this analysis, each tissue-specific gene, each pancRNA-partnered tissue-specific gene, and each pancRNA-lacking tissue-specific gene was utilized (TSI > 0.9). The standard error of the mean across the regions is shown as semi-transparent shading around the mean curve. (A) Cerebellum-specific genes. (B) Heart-specific genes. (C) Kidney-specific genes. (D) Liver-specific genes. (PDF 617 kb)

Additional file 6: Figure S4. Expression levels of tissue-specific genes, of pancRNA-partnered tissue-specific genes, and of pancRNA-lacking tissue-specific genes (TSI > 0.9). *** *P* <0.001; Error bars indicate the first and third quartiles. (PDF 69 kb)

Additional file 7: Table S3. The percentages of promoter regions overlapping with CpG islands. (XLS 36 kb)

Additional file 8: Figure S5. The DNA motifs enriched at the immediately upstream regions of the TSS of pancRNA-partnered genes. The top three most statistically significant motifs and the E-value of each motif are shown for the five species. (PDF 362 kb)

Additional file 9: Table S4. Exclusivity of C-rich and G-rich motifs at the immediately upstream regions of TSS of pancRNA-partnered genes. (XLS 27 kb)

Additional file 10: Figure S6. Hierarchical clustering of mRNA and pancRNA expression profiles. Dendrogram represents the average linkage hierarchical clustering of mRNA (A) and pancRNA (B) expression profiles of the five tissues in the five species. The distance between data was computed as 1 − ρ, where ρ is the Spearman correlation coefficient. (PDF 62 kb)

Additional file 11: Figure S7. Diversity of conserved pancRNA expression profile of the five tissues in the five species. Hierarchical clustering and symmetrical heat map of Spearman correlation coefficients of conserved pancRNA (A) and their corresponding mRNA (B) expression profiles. Samples are colored according to the tissues and the species. (PDF 301 kb)

Additional file 12: Table S5. Species-specific pancRNA-partnered genes. (XLS 64 kb)

## Abbreviations

Cbellum: Cerebellum
CDS: Coding sequence regions
ChIP-seq: Chromatin immunoprecipitation together with DNA sequencing
Cortex: Cerebral cortex
H3K27ac: Acetylated lysine 27 of histone H3
H3K4: Lysine 4 of histone 3
H3K4me1: Mono-methylated H3K4
H3K4me3: Tri-methylated H3K4
H3K9: Lysine 9 of histone 3
lncRNA: Long intergenic non-coding RNA
ncRNA: Non-coding RNA
pancRNA: Promoter-associated non-coding RNA
RNA-seq: RNA sequencing
RPM: Read count per million mapped reads
SAT: Sense-antisense transcript
TSI: Tissue-specificity index
TSS: Transcription start site
