# Supplementary figures and images for: Evolutionary acquisition of promoter-associated non-coding RNA (pancRNA) repertoires diversifies species-dependent gene activation mechanisms in mammals
Source: BMC Genomics. 2017 Apr 7;18:285. doi: 10.1186/s12864-017-3662-1 (PMC5383967; doi:10.1186/s12864-017-3662-1)

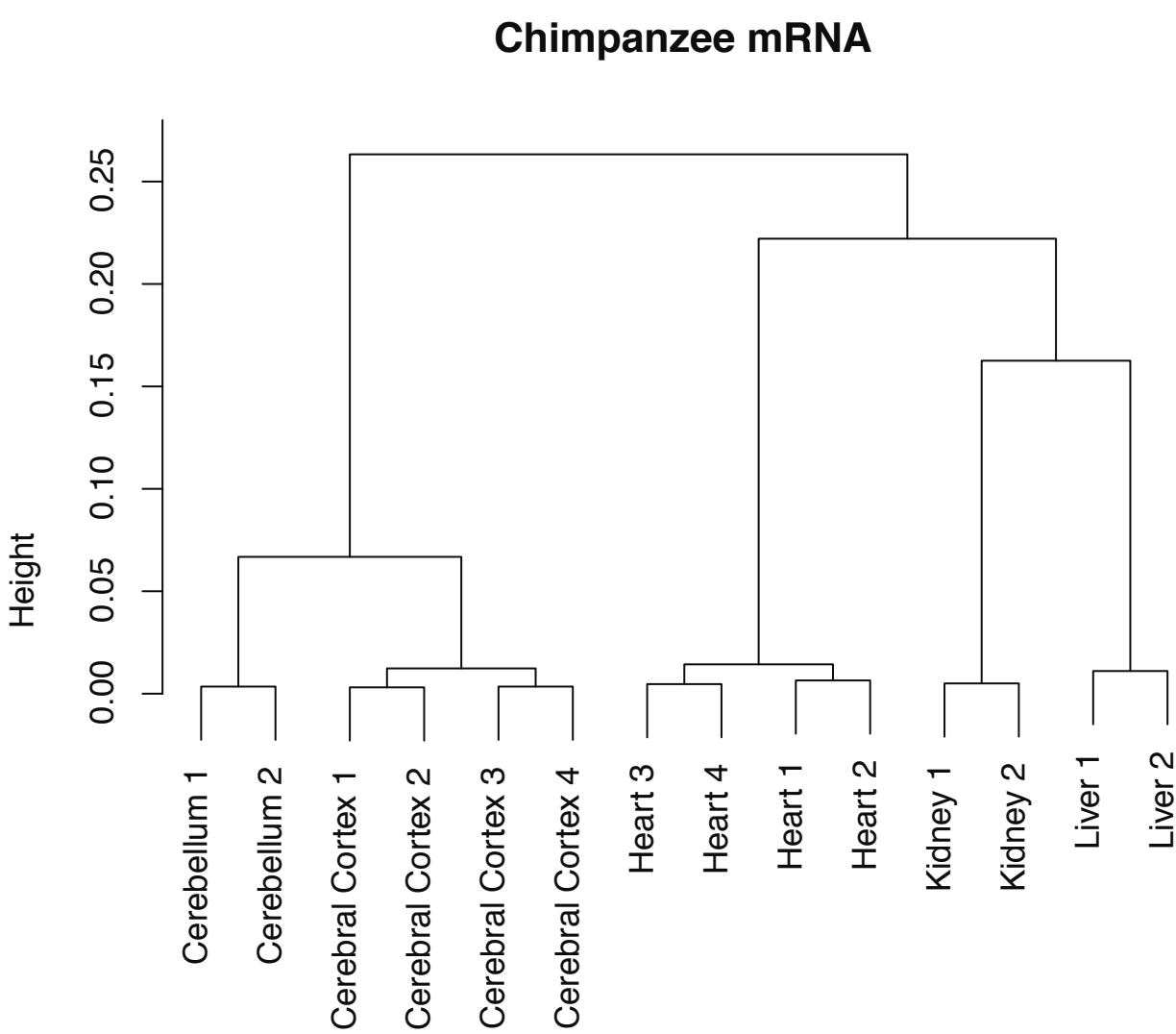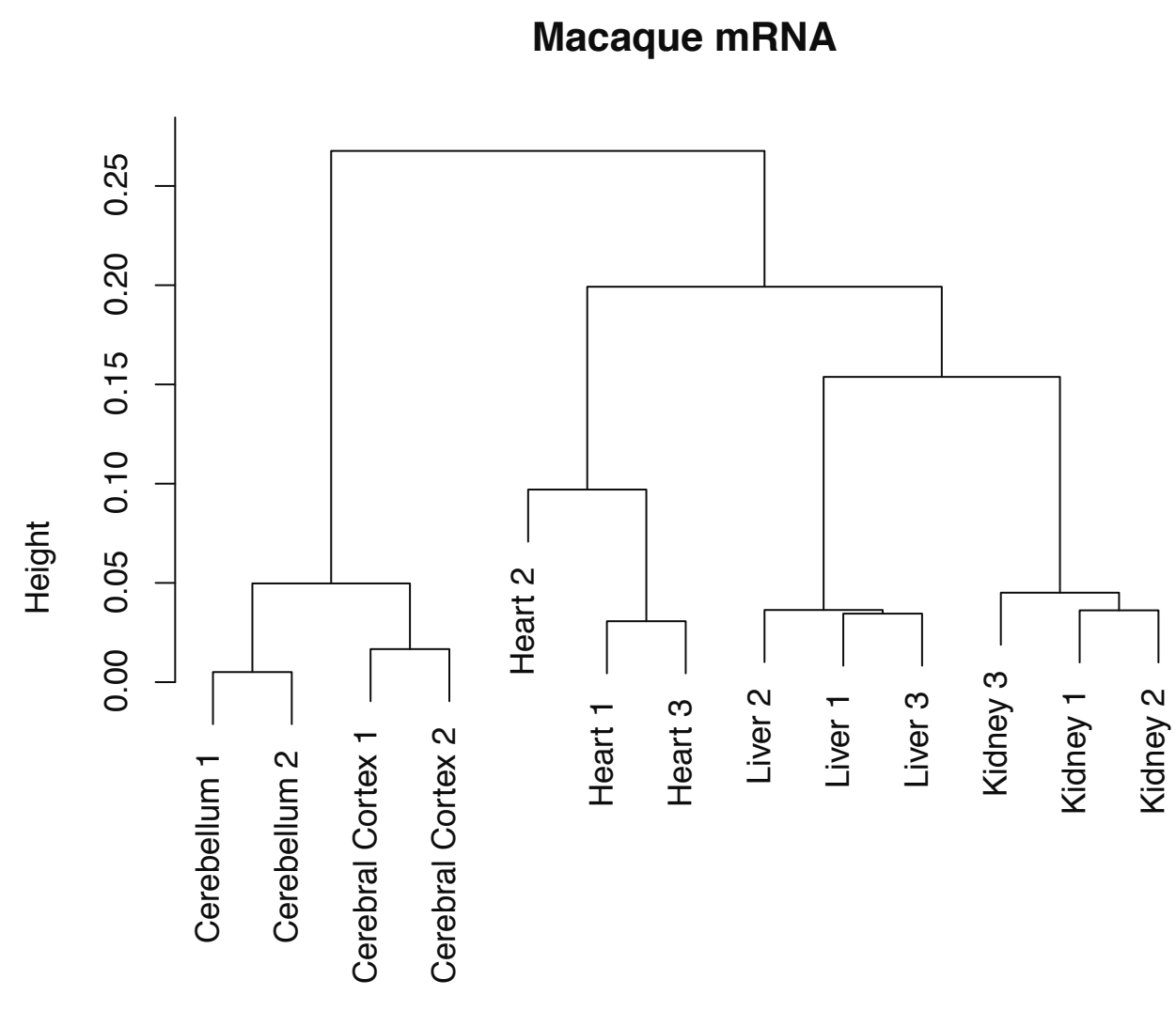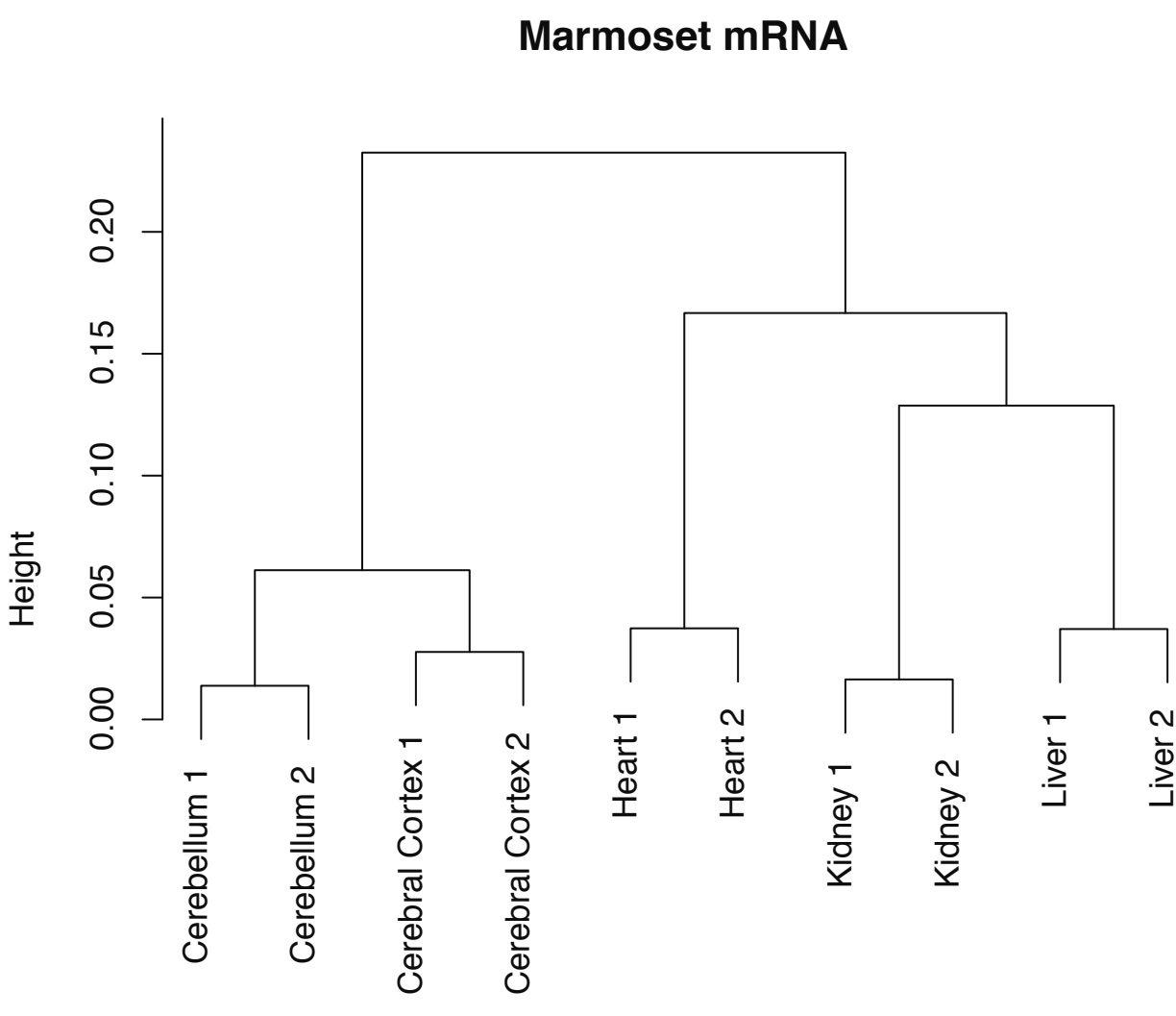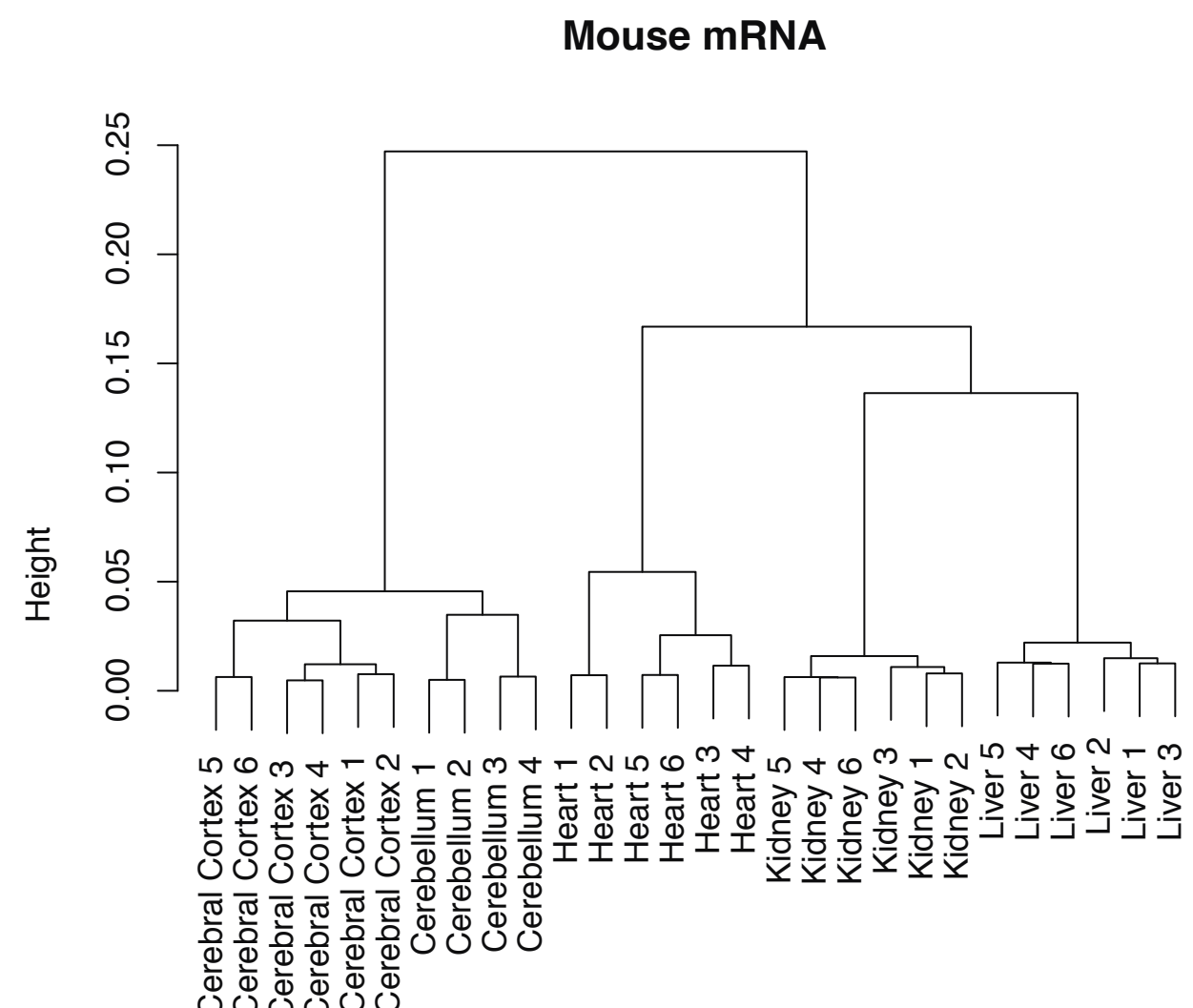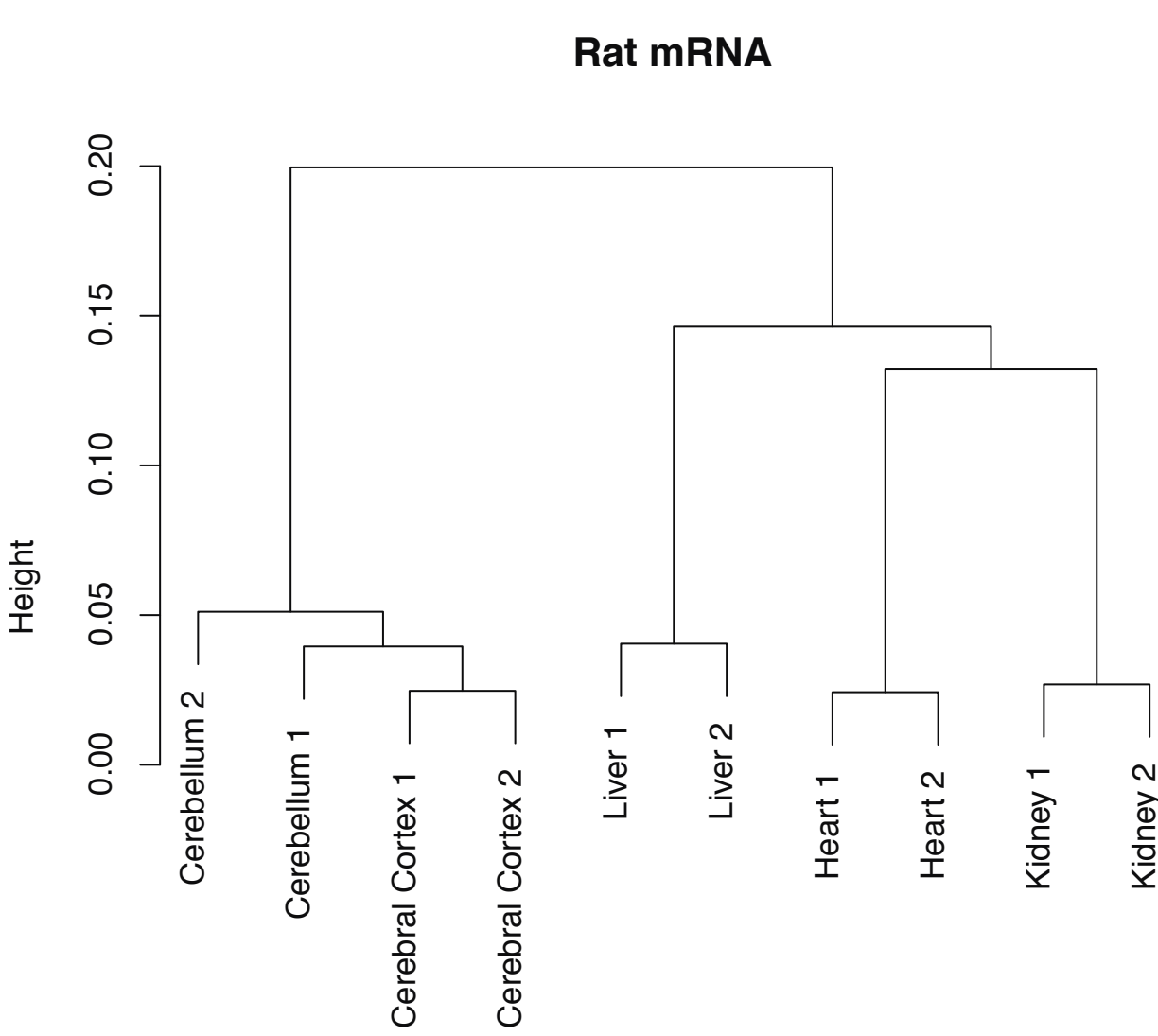

**Figure S1**

Supplement: Supplementary file 3 — Hierarchical clustering of directional RNA-seq data. Dendrogram represents average linkage hierarchical clustering of directional RNA-seq data based on the mRNA expression profiles in each of the five species. The distance between data was computed as 1 − ρ, where ρ is the Spearman correlation coefficient. Note that the gene expression profiles of 16-week-old mice (mouse cerebral cortex sample #1-4; home-made RNA-seq data) and 8-week-old mice (mouse cerebral cortex sample #5-6; RNA-seq data from the mouse ENCODE project) are quite similar to each other. (PDF 60 kb) [file 12864_2017_3662_MOESM3_ESM.pdf]

Tissue-specificity index

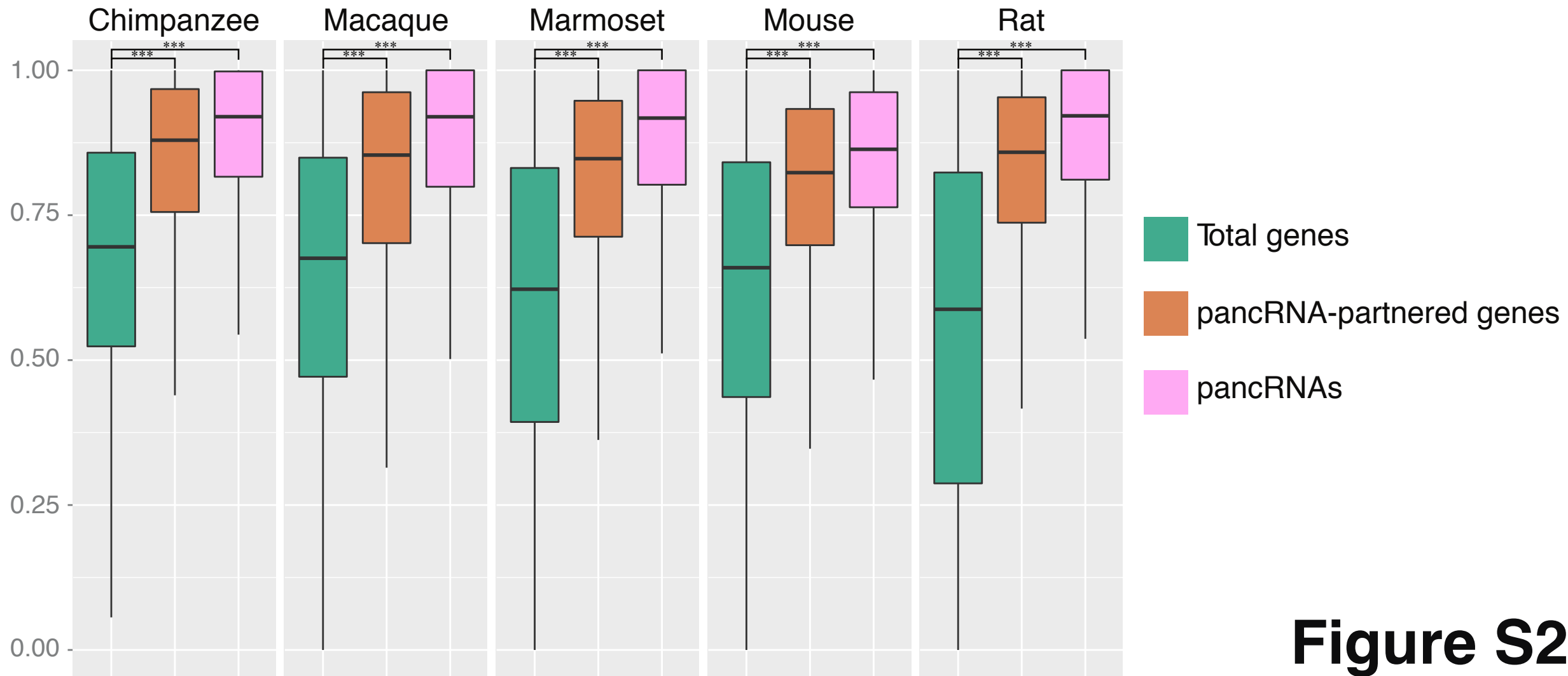

**Figure S2**

Supplement: Supplementary file 4 — Tissue-specificity index of total protein-coding genes, of pancRNA-partnered genes, and of pancRNAs. *** P <0.001; Error bars indicate the first and third quartiles. (PDF 66 kb) [file 12864_2017_3662_MOESM4_ESM.pdf]

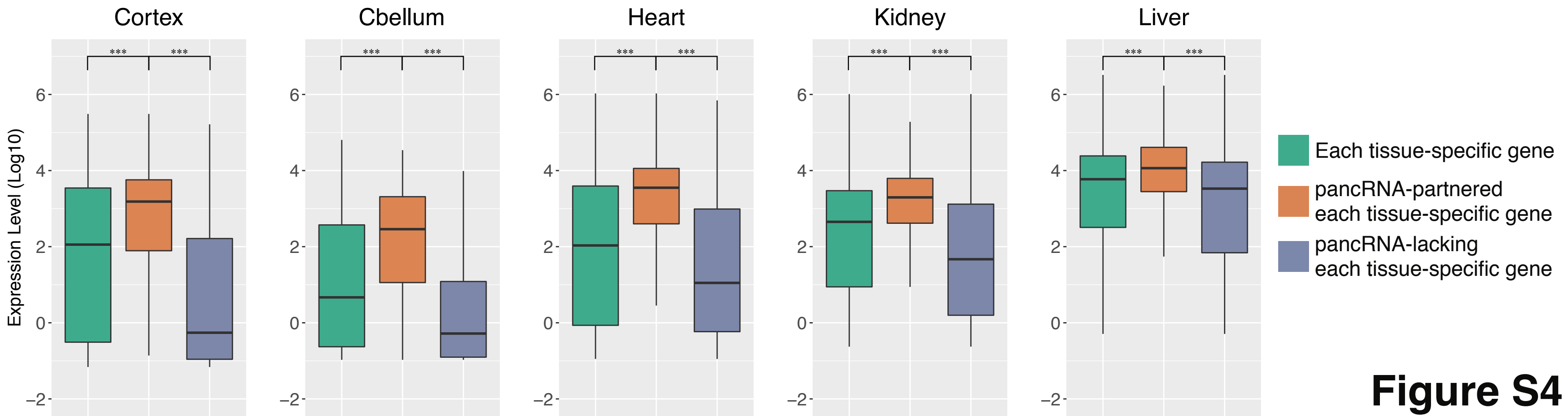

**Figure S4**

Supplement: Supplementary file 6 — Expression levels of tissue-specific genes, of pancRNA-partnered tissue-specific genes, and of pancRNA-lacking tissue-specific genes (TSI > 0.9). *** P <0.001; Error bars indicate the first and third quartiles. (PDF 69 kb) [file 12864_2017_3662_MOESM6_ESM.pdf]

**Chimpanzee**

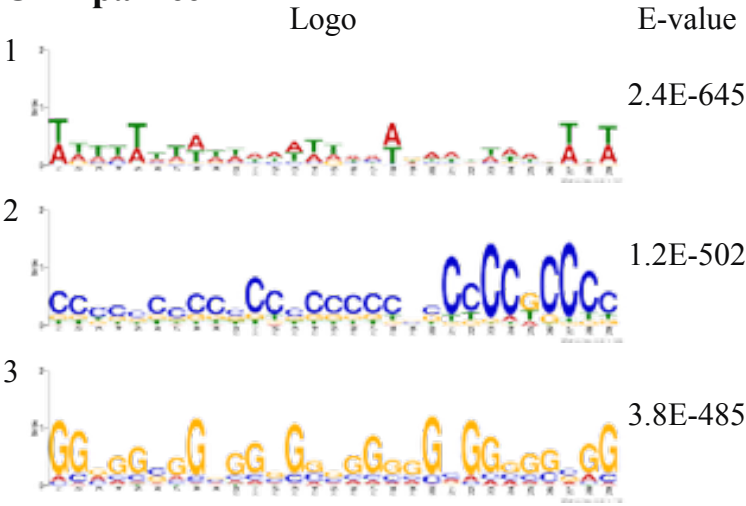

**Macaque**

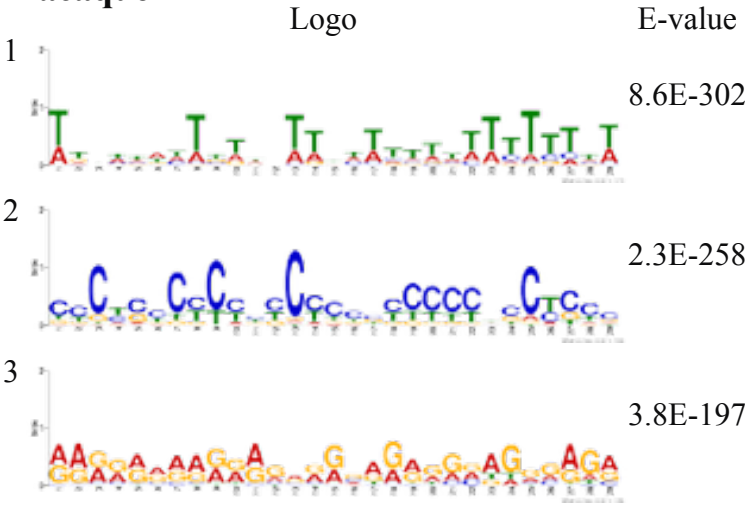

**Marmoset**

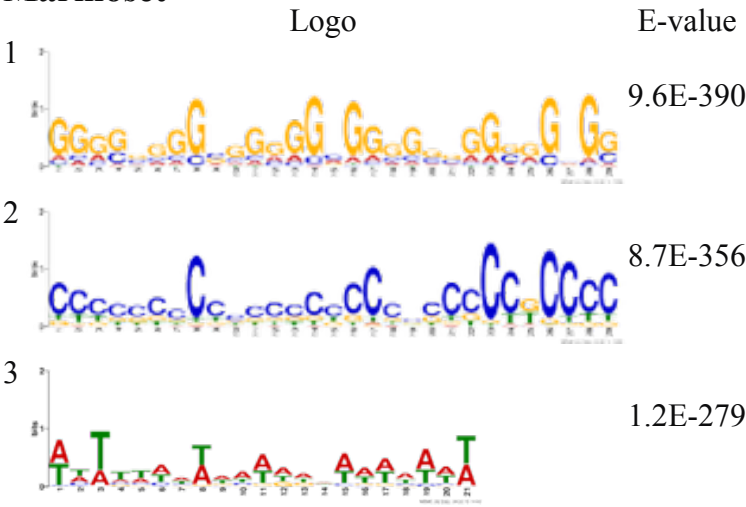

**Mouse**

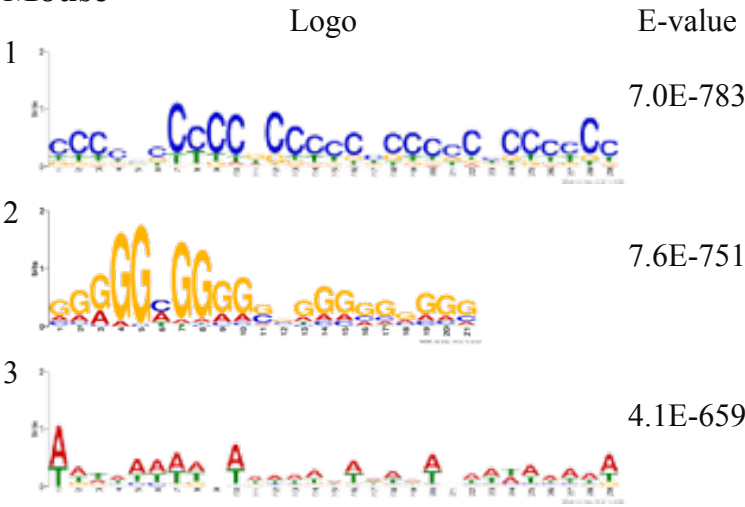

**Rat**

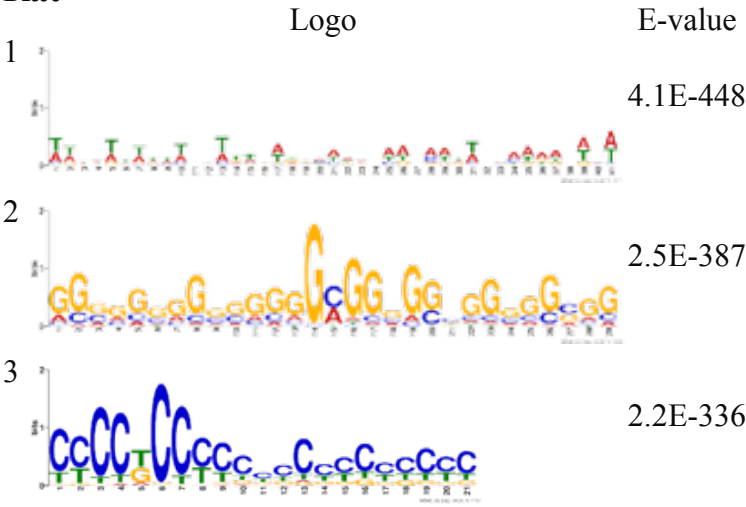

**Figure S5**

Supplement: Supplementary file 8 — The DNA motifs enriched at the immediately upstream regions of the TSS of pancRNA-partnered genes. The top three most statistically significant motifs and the E-value of each motif are shown for the five species. (PDF 362 kb) [file 12864_2017_3662_MOESM8_ESM.pdf]

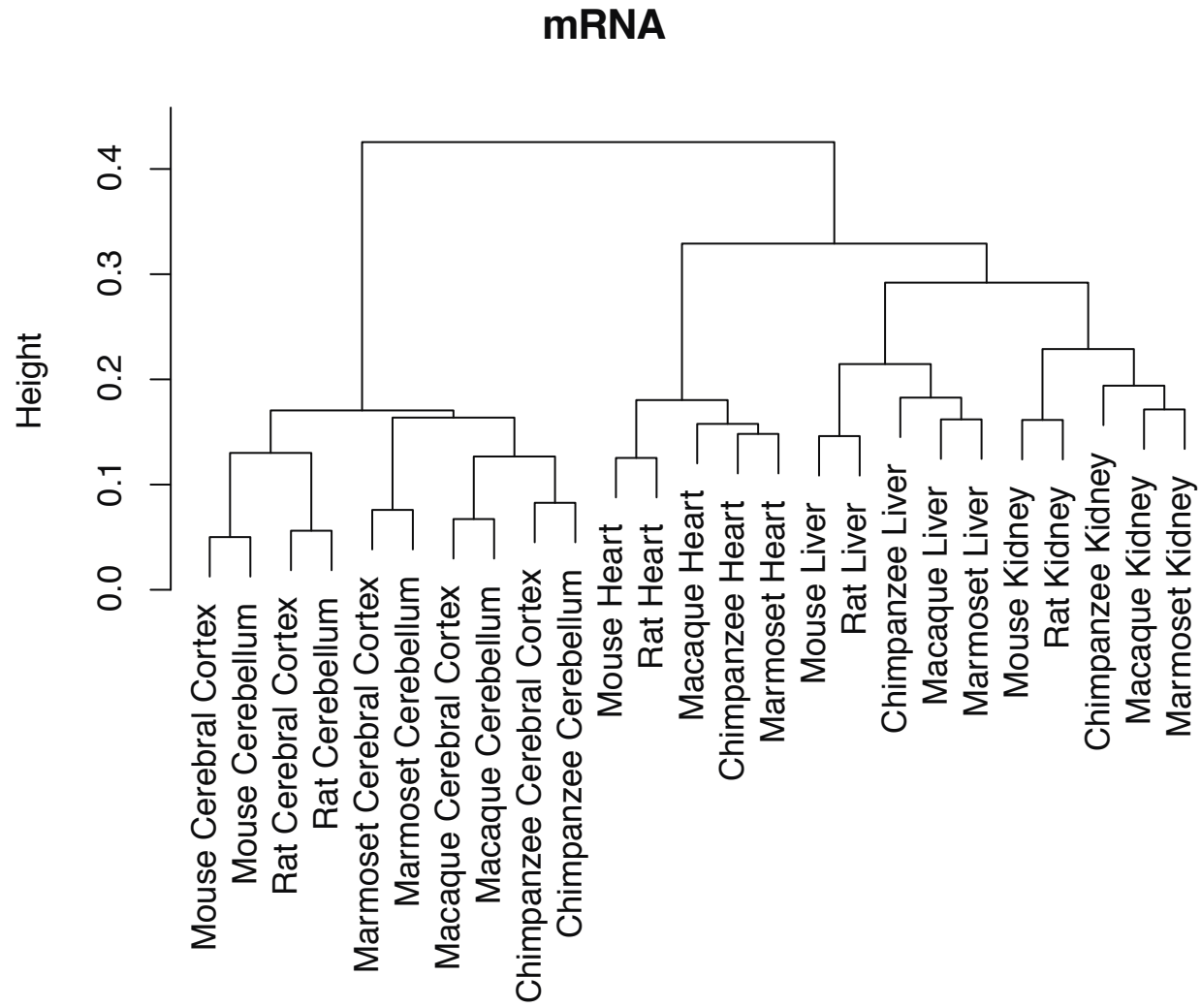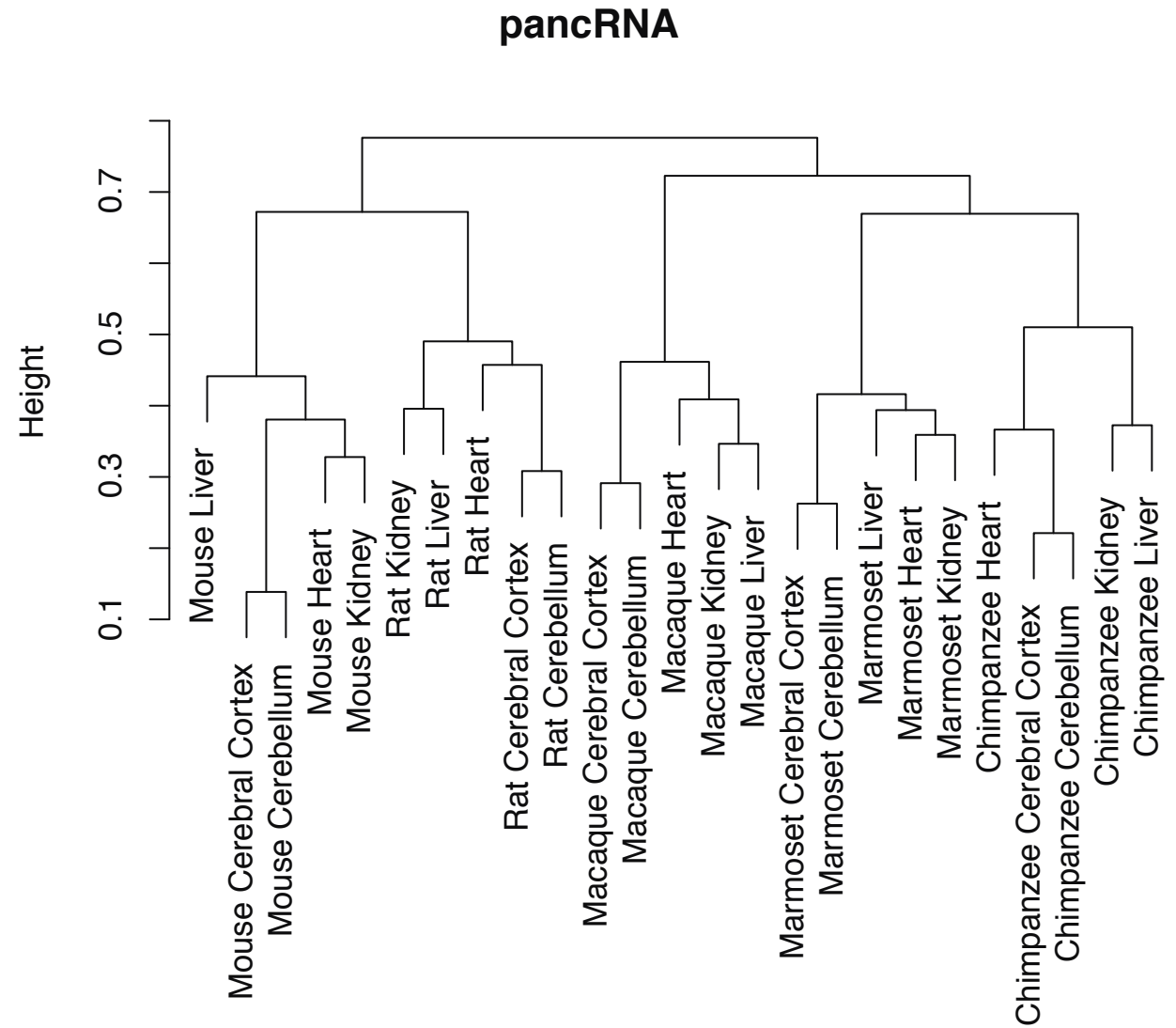

**Figure S6**

Supplement: Supplementary file 10 — Hierarchical clustering of mRNA and pancRNA expression profiles. Dendrogram represents the average linkage hierarchical clustering of mRNA (A) and pancRNA (B) expression profiles of the five tissues in the five species. The distance between data was computed as 1 − ρ, where ρ is the Spearman correlation coefficient. (PDF 62 kb) [file 12864_2017_3662_MOESM10_ESM.pdf]

**A**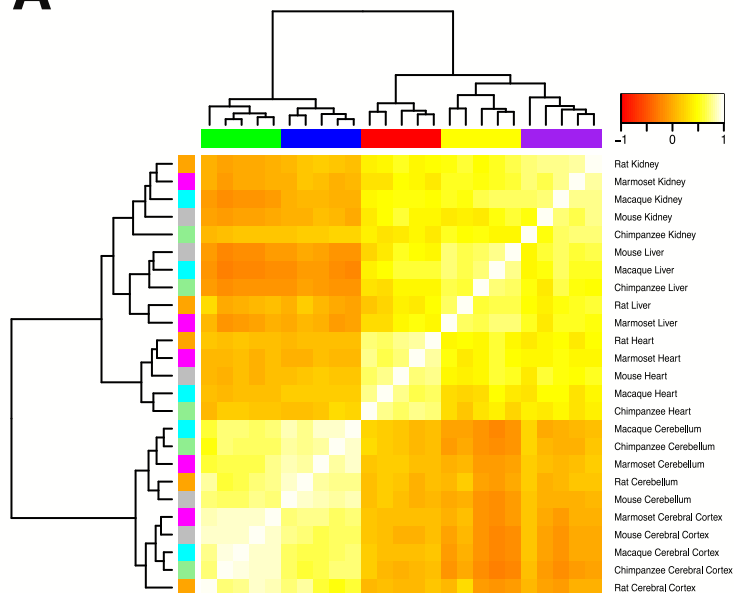**B**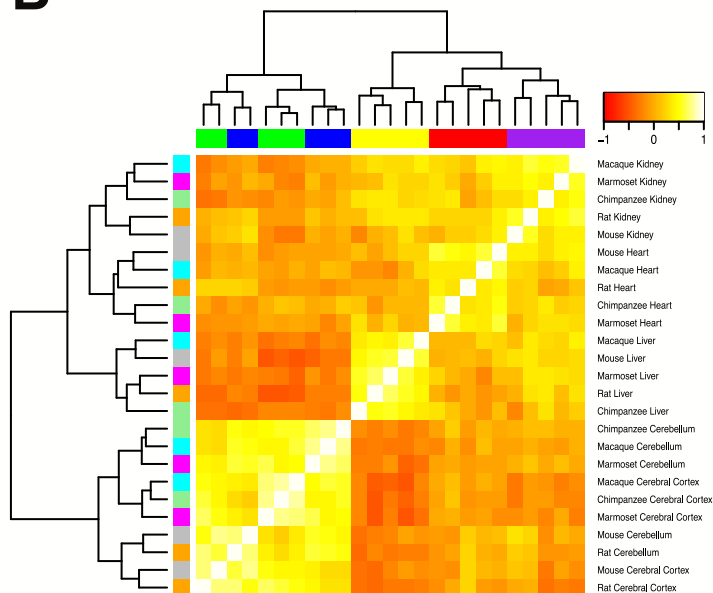

# Figure S7

Supplement: Supplementary file 11 — Diversity of conserved pancRNA expression profile of the five tissues in the five species. Hierarchical clustering and symmetrical heat map of Spearman correlation coefficients of conserved pancRNA (A) and their corresponding mRNA (B) expression profiles. Samples are colored according to the tissues and the species. (PDF 301 kb) [file 12864_2017_3662_MOESM11_ESM.pdf]
