# Supplementary material for: Evolutionary acquisition of promoter-associated non-coding RNA (pancRNA) repertoires diversifies species-dependent gene activation mechanisms in mammals
Source: BMC Genomics. 2017 Apr 7;18:285. doi: 10.1186/s12864-017-3662-1 (PMC5383967; doi:10.1186/s12864-017-3662-1)
Supplement: Supplementary file 5 — Histone modification across the regions around TSSs of each tissue-specific gene. RPM (Read count per million mapped reads) derived from ChIP-seq data (H3K4me1, H3K4me3, and H3K27ac) of mouse cerebral cortex (Cortex), cerebellum (Cbellum), heart, kidney, and liver across the regions around TSSs (−2,000 bp to +2,000 bp relative to TSS). In this analysis, each tissue-specific gene, each pancRNA-partnered tissue-specific gene, and each pancRNA-lacking tissue-specific gene was utilized (TSI > 0.9). The standard error of the mean across the regions is shown as semi-transparent shading around the mean curve. (A) Cerebellum-specific genes. (B) Heart-specific genes. (C) Kidney-specific genes. (D) Liver-specific genes. (PDF 617 kb) [file 12864_2017_3662_MOESM5_ESM.pdf]

Max Cbellum

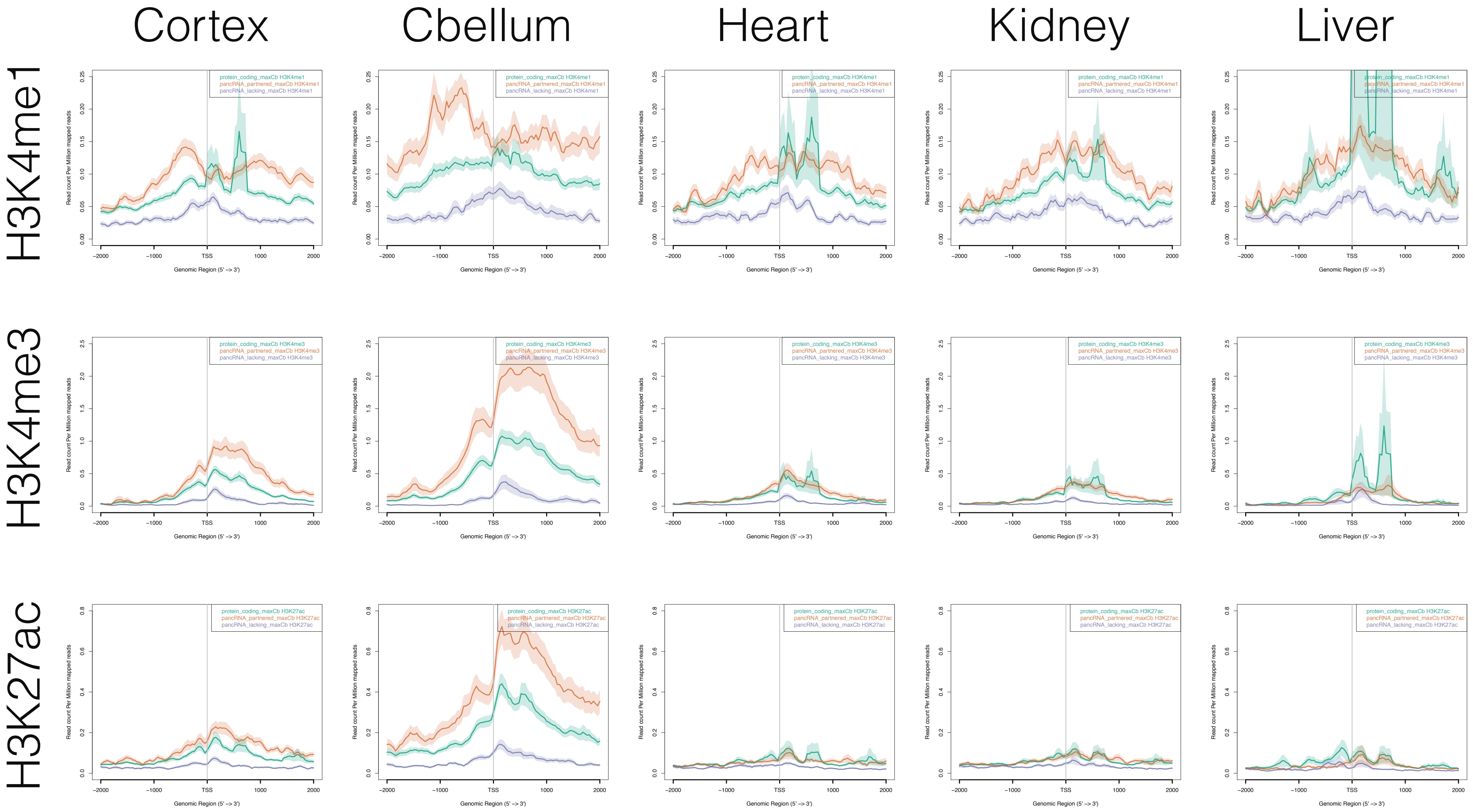

Figure S3A

Max Heart

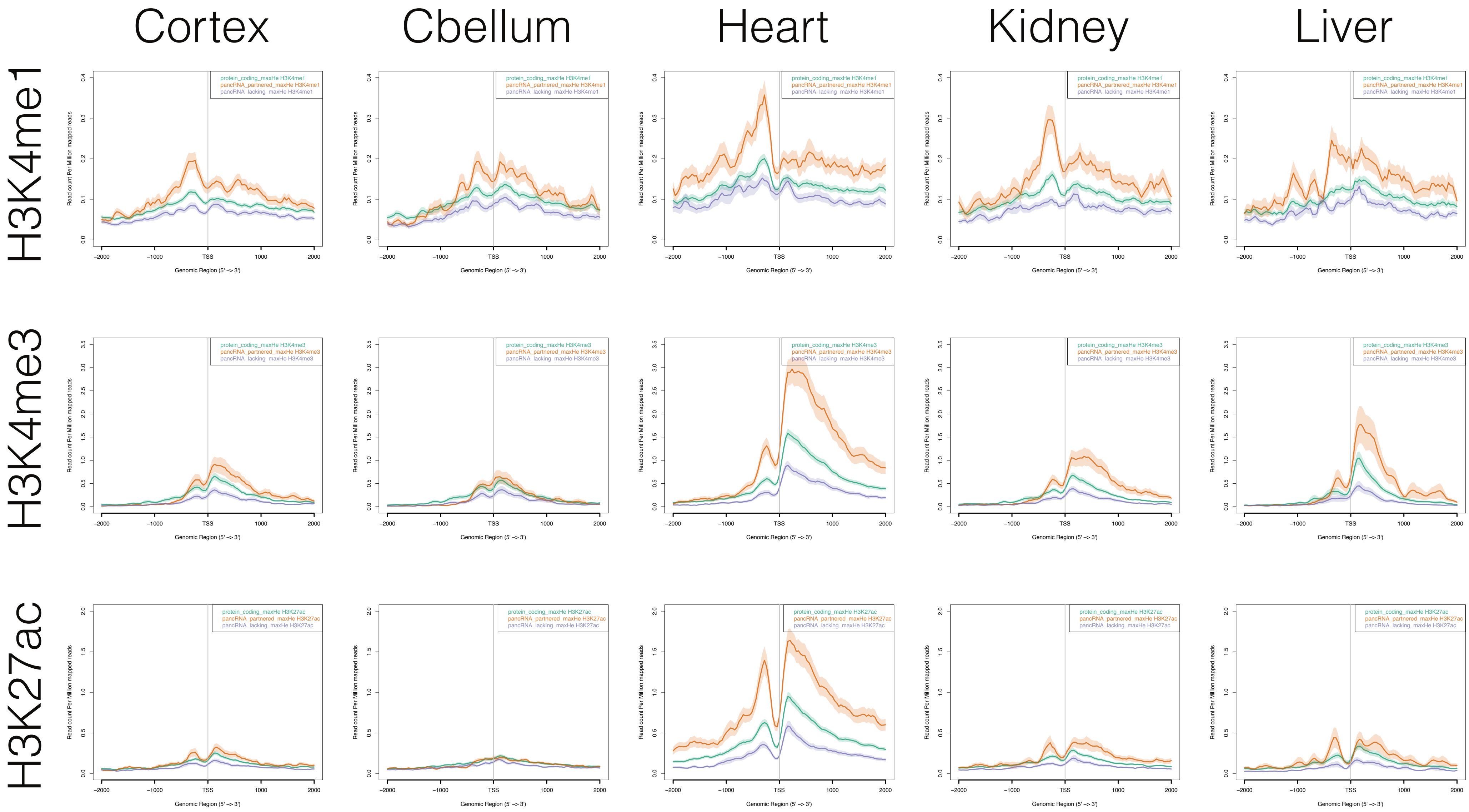

Figure S3B

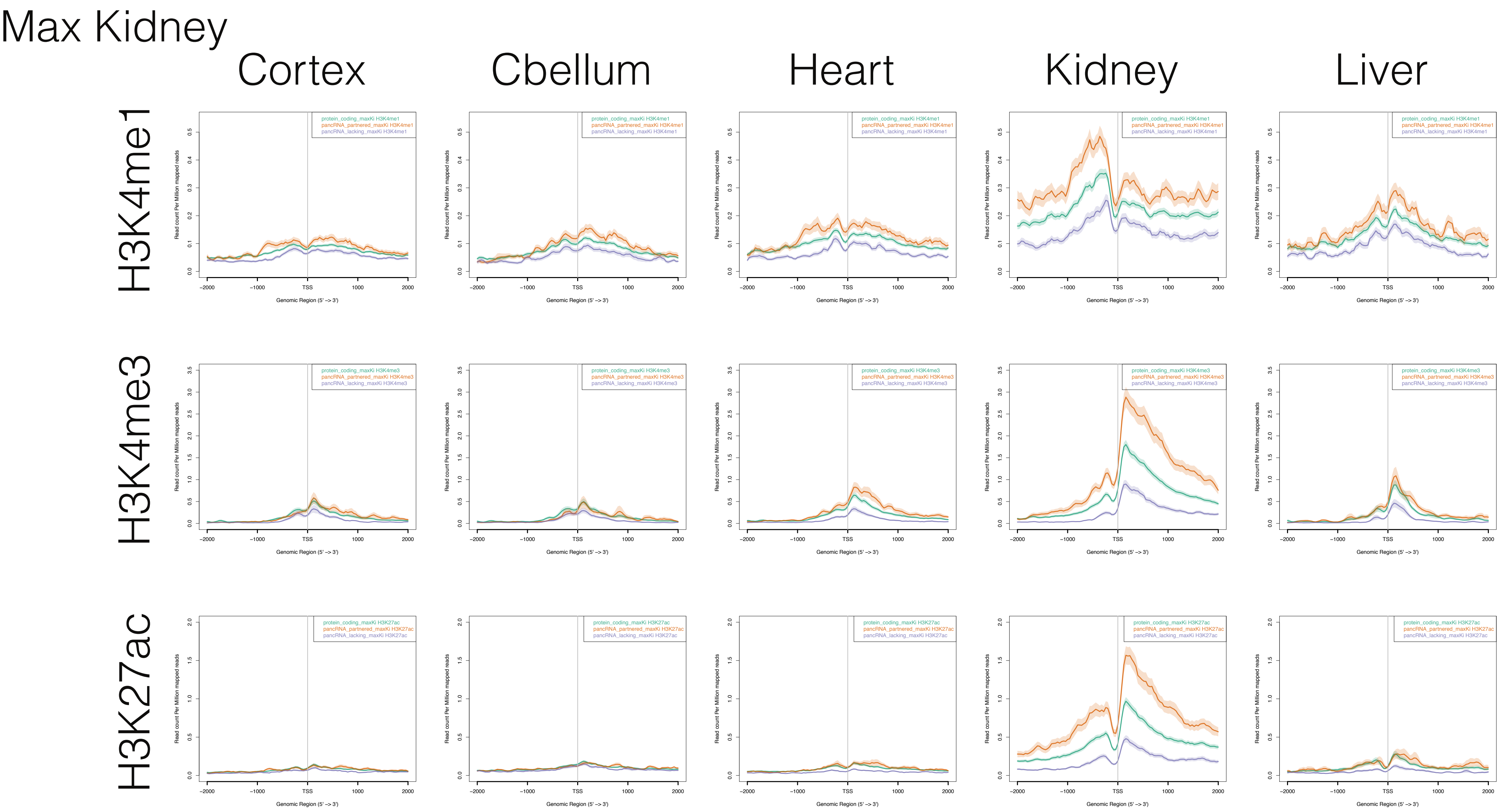
Max Kidney
Cortex
Cbellum
Heart
Kidney
Liver
H3K4me1
H3K4me3
H3K27ac
protein_coding_maxKi H3K4me1
pancRNA_partnered_maxKi H3K4me1
pancRNA_lacking_maxKi H3K4me1
protein_coding_maxKi H3K4me3
pancRNA_partnered_maxKi H3K4me3
pancRNA_lacking_maxKi H3K4me3
protein_coding_maxKi H3K27ac
pancRNA_partnered_maxKi H3K27ac
pancRNA_lacking_maxKi H3K27ac
Read count Per Million mapped reads
Genomic Region (5' -> 3')
TSS

Figure S3C

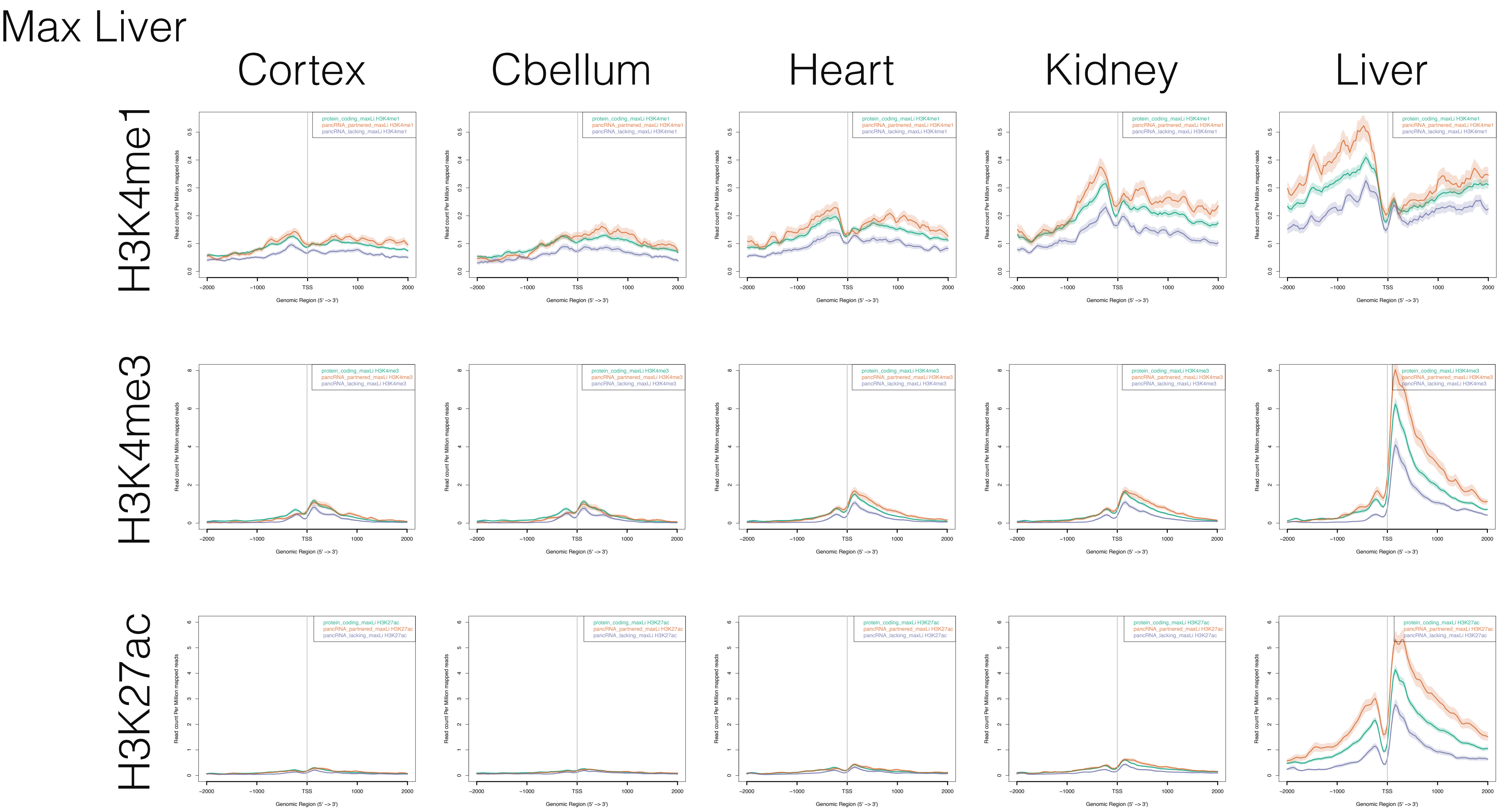
Max Liver
Cortex
Cbellum
Heart
Kidney
Liver
H3K4me1
H3K4me3
H3K27ac
protein_coding_maxLi H3K4me1
pancRNA_partnered_maxLi H3K4me1
pancRNA_lacking_maxLi H3K4me1
protein_coding_maxLi H3K4me3
pancRNA_partnered_maxLi H3K4me3
pancRNA_lacking_maxLi H3K4me3
protein_coding_maxLi H3K27ac
pancRNA_partnered_maxLi H3K27ac
pancRNA_lacking_maxLi H3K27ac
Read count Per Million mapped reads
Genomic Region (5' -> 3')
-2000
-1000
TSS
1000
2000

Figure S3D
